# Benthic primary production in an upwelling-influenced coral reef, Colombian Caribbean

**DOI:** 10.7717/peerj.554

**Published:** 2014-09-02

**Authors:** Corvin Eidens, Elisa Bayraktarov, Torsten Hauffe, Valeria Pizarro, Thomas Wilke, Christian Wild

**Affiliations:** 1 Department of Animal Ecology & Systematics, Justus Liebig University Giessen, Germany; 2 Coral Reef Ecology Group (CORE), Leibniz Center for Tropical Marine Ecology, Bremen, Germany; 3 Center of Excellence in Marine Sciences (CEMarin), Santa Marta, Colombia; 4 Faculty of Biology and Chemistry, University of Bremen, Germany

**Keywords:** O_2_ flux, Ecosystem productivity, Resilience, Coastal upwelling, Seasonal variation, Tayrona National Natural Park

## Abstract

In Tayrona National Natural Park (Colombian Caribbean), abiotic factors such as light intensity, water temperature, and nutrient availability are subjected to high temporal variability due to seasonal coastal upwelling. These factors are the major drivers controlling coral reef primary production as one of the key ecosystem services. This offers the opportunity to assess the effects of abiotic factors on reef productivity. We therefore quantified primary net (*P_n_*) and gross production (*P_g_*) of the dominant local primary producers (scleractinian corals, macroalgae, algal turfs, crustose coralline algae, and microphytobenthos) at a water current/wave-exposed and-sheltered site in an exemplary bay of Tayrona National Natural Park. A series of short-term incubations was conducted to quantify O_2_ fluxes of the different primary producers during non-upwelling and the upwelling event 2011/2012, and generalized linear models were used to analyze group-specific O_2_ production, their contribution to benthic O_2_ fluxes, and total daily benthic O_2_ production. At the organism level, scleractinian corals showed highest *P_n_* and *P_g_* rates during non-upwelling (16 and 19 mmol O_2_ m^−2^ specimen area h^−1^), and corals and algal turfs dominated the primary production during upwelling (12 and 19 mmol O_2_ m^−2^ specimen area h^−1^, respectively). At the ecosystem level, corals contributed most to total *P_n_* and *P_g_* during non-upwelling, while during upwelling, corals contributed most to *P_n_* and *P_g_* only at the exposed site and macroalgae at the sheltered site, respectively. Despite the significant spatial and temporal differences in individual productivity of the investigated groups and their different contribution to reef productivity, differences for daily ecosystem productivity were only present for *P_g_* at exposed with higher O_2_ fluxes during non-upwelling compared to upwelling. Our findings therefore indicate that total benthic primary productivity of local autotrophic reef communities is relatively stable despite the pronounced fluctuations of environmental key parameters. This may result in higher resilience against anthropogenic disturbances and climate change and Tayrona National Natural Park should therefore be considered as a conservation priority area.

## Introduction

The majority of ecosystems depend on primary production. Photoautotrophs convert light energy into chemical energy by photosynthesis, creating the energetic base of most food webs in terrestrial as well as aquatic environments (Chapin et al., 2011; Valiela, 1995). Among other coastal ecosystems such as mangrove forests, seagrass beds, salt marshes, and kelp forests, coral reefs belong to the most productive ecosystems in the world (Gattuso, Frankignoulle & Wollast, 1998; Hatcher, 1988). Productivity investigation on coral reefs started in the mid-20th century (Odum & Odum, 1955; Sargent & Austin, 1949) and nowadays, coral reefs are among the best understood marine benthic communities in terms of primary production (Gattuso, Frankignoulle & Wollast, 1998; Hatcher, 1988; Hatcher, 1990; Kinsey, 1985).

It was long assumed that coral reef productivity is relatively balanced as tropical coral reefs typically thrive under relatively stable abiotic conditions (Hubbard, 1996; Kleypas, McManus & Menez, 1999; Sheppard, Davy & Pilling, 2009), including light (Achituv & Dubinsky, 1990; Darwin, 1842; Falkowski, Jokiel & Kinzie, 1990), water temperature (Coles & Fadlallah, 1991; Dana, 1843; Veron, 1995), salinity (Andrews & Pickard, 1990; Coles & Jokiel, 1992), and inorganic nutrient availability (D’Elia & Wiebe, 1990; Szmant, 1997).

Nevertheless, coral reefs also occur in seasonal upwelling-affected regions such as the Arabian Sea off Oman (Glynn, 1993), the Eastern Tropical Pacific off Panamá and Costa Rica (Cortés & Jiménez, 2003; Glynn & Stewart, 1973), and the Colombian Caribbean (Geyer, 1969). Whereas several studies focused on the seasonality of benthic primary production in coral reefs at different latitudes (Adey & Steneck, 1985; Falter et al., 2012; Kinsey, 1985), variability in primary production of seasonal upwelling-affected coral reefs remains largely unknown.

The Tayrona National Natural Park (TNNP) at the Caribbean coast of Colombia is highly influenced by the Southern Caribbean upwelling system (Andrade & Barton, 2005; Rueda-Roa & Muller-Karger, 2013), causing seasonal fluctuations in water temperature, salinity, and inorganic nutrient concentrations, among others (Table 1, see also Bayraktarov, Pizarro & Wild, 2014). Here, the abundance and community composition of benthic algae were shown to exhibit upwelling-related seasonality (Diaz-Pulido & Garzón-Ferreira, 2002; Eidens et al., 2012). The area thereby provides an excellent opportunity to investigate the effects of seasonal coastal upwelling events on the key ecosystem service productivity in coral reefs under changing *in situ* conditions. The results of a preliminary study conducted by Eidens et al. (2012) indicated that benthic primary production in TNNP differed between the upwelling in 2010/2011 and the consecutive non-upwelling season, suggesting a generally positive effect of upwelling conditions on major benthic autotrophs in the area. However, after unusually strong El Niño-Southern Oscillation (ENSO) events in 2010, the area experienced a moderate coral bleaching before the upwelling in 2010/2011 (Bayraktarov et al., 2013; Hoyos et al., 2013), and productivity measurements during upwelling in 2010/2011 may not be representative. To test for patterns in benthic primary production during a typical seasonal cycle, we here quantified benthic primary production before and at the end of the upwelling event in 2011/2012 (hereafter referred to as non-upwelling and upwelling, respectively). To allow for comparisons of productivity between investigated groups, we further estimated surface area-specific productivity rates as suggested by Naumann et al. (2013) and analyzed the data using generalized linear models.

**Table 1:** Seasonality in water temperature, salinity and nitrate availability in Gayraca Bay. Mean values (±SD) at the exposed and sheltered sites and a water depth of 10 m for upwelling (December–April) and non-upwelling (May–November) periods from 2010–2013.

Variables	Non-upwelling		Upwelling		Range
	Exposed	Sheltered	Exposed	Sheltered	
Temperature (°C)	28.5 ± 1.7	28.7 ± 1.7	25.7 ± 2.6	25.7 ± 2.5	20.5–30.0
Salinity	35.3 ± 1.5	35.3 ± 1.2	37.1 ± 1.1	37.0 ± 0.8	32.6–38.5
Nitrate (µmol L^−1^)	0.26 ± 0.20	0.32 ± 0.16	1.31 ± 0.95	1.34 ± 0.99	nd–3.59

**Notes.**

nd, below detection level.

Therefore, the goals of the study were to (1) identify dominant functional groups of benthic primary producers and their relative benthic cover at a current/wave-exposed (EXP) and -sheltered (SHE) site in one exemplary bay of TNNP, (2) quantify O_2_ fluxes of all dominant benthic primary producers and apply 3D surface area estimations, and hence (3) estimate the specific contribution of each group to total benthic O_2_ fluxes.

## Materials and Methods

### Study site and sampling seasons

**Figure 1:**
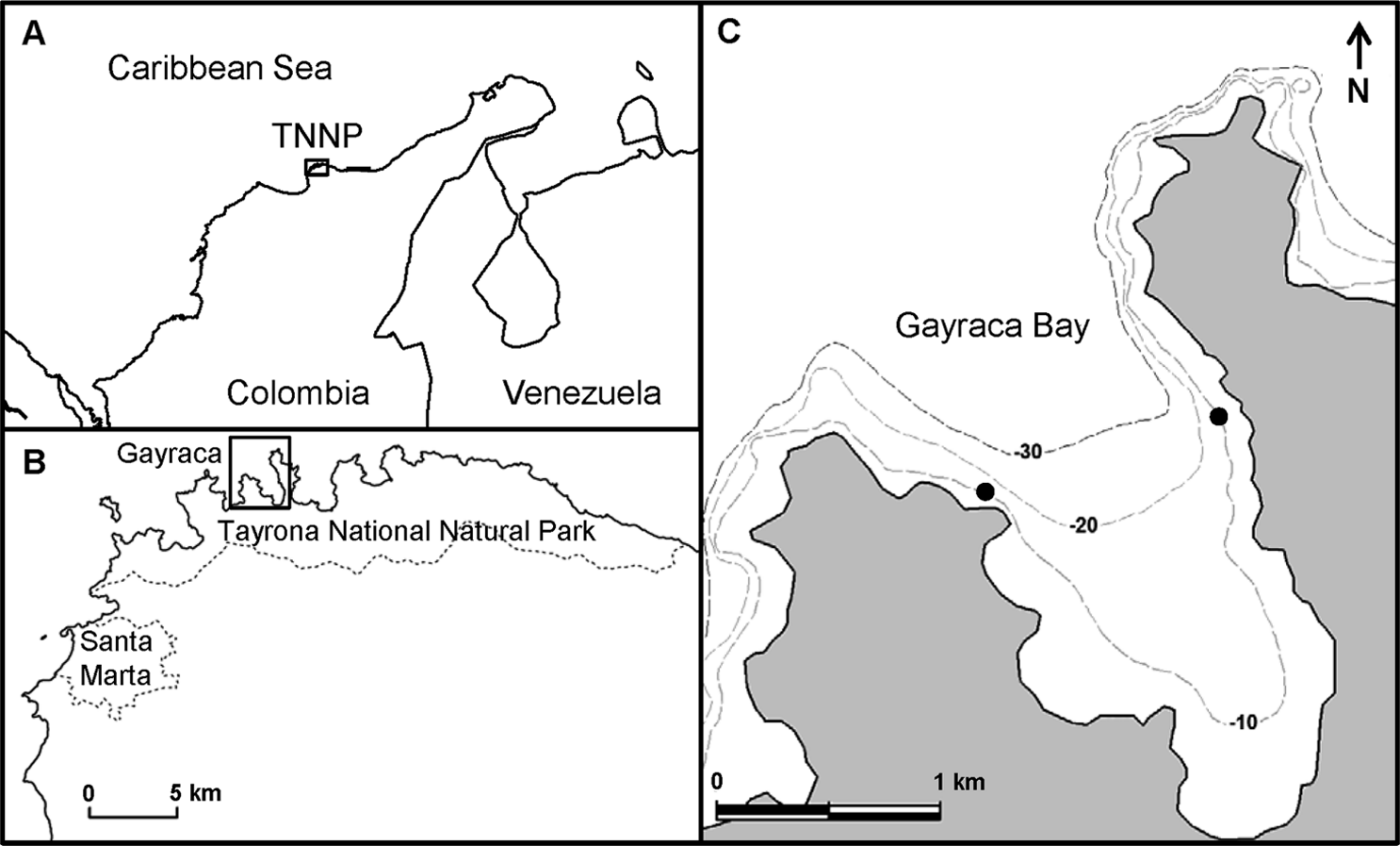
Location of study sites. (A) Location of Tayrona National Natural Park (TNNP) at the Caribbean coast of Colombia. (B) Location of Gayraca Bay within TNNP (dashed lines–national park border and expansion of the city of Santa Marta. (C) Gayraca Bay. The investigation sites at the current-exposed western part and the sheltered north-eastern part are indicated by black dots (dashed lines–isobaths). Source of map: INVEMAR (2012).

This study was conducted in Gayraca Bay (11.33°N, 74.11°W), one of several smaller bays in TNNP, located near the city of Santa Marta (Fig. 1). The continental shelf in the area is relatively narrow due to the proximity to the Sierra Nevada de Santa Marta–the world’s highest coastal mountain range. The TNNP contains small fringing coral reefs reaching to a water depth of ∼30 m (Garzón-Ferreira, 1998; Garzón-Ferreira & Cano, 1991). The region is subjected to strong seasonality caused by the Caribbean Low-Level Jet of northeast (NE) trade winds (Andrade & Barton, 2005; Salzwedel & Müller, 1983), resulting in two major seasons; a dry season from December to April and a rainy season from May to November (Garzón-Ferreira, 1998; Salzwedel & Müller, 1983). Whereas the rainy season (non-upwelling) is characterized by low wind velocities (mean 1.5 m s^−1^) (Garzón-Ferreira, 1998) and high precipitation (>80% of the annual rainfall) (Salzwedel & Müller, 1983), during the dry season (upwelling), strong winds prevail (mean 3.5 m s^−1^, max 30 m s^−1^) (Herrmann, 1970; Salzwedel & Müller, 1983) resulting in a seasonal coastal upwelling. The upwelling-related changes in key water parameters are well characterized by the comprehensive study of Bayraktarov, Pizarro & Wild (2014). During upwelling, water temperature can decrease to 20 °C while salinity and nitrate availability increase up to 39 and 3.59 µmol L^−1^, respectively (Table 1). Water currents triggered by prevailing winds predominantly move from NE to SW, and a clear gradient in wave exposure between the exposed western (EXP) and -sheltered northeastern (SHE) sides of the bay can be observed (Bayraktarov, Bastidas-Salamanca & Wild, 2014; Werding & Sánchez, 1989).The study was carried out during non-upwelling in 2011 (1st November–2nd December 2011) and during the consecutive upwelling event (20th March–29th March 2012), allowing for the investigation of the influence of seasonality on benthic primary production.

### Benthic assessment

For the assessment of benthic community structure, the dominant groups of benthic primary producers and the percentage of benthic cover were identified at EXP and SHE prior to primary production measurements using line point intercept transects at a water depth of 10 m (50 m length, *n* = 3), modified from Hodgson et al. (2004). Benthic cover was monitored at 0.5 m intervals directly below the measurement points (101 data points per transect). The dominant benthic autotrophs at the study sites consisted of scleractinian corals, frondose macroalgae, algal turfs (multispecific assemblage of primarily filamentous algae of up to 1 cm height, *sensu* Steneck (1988)), crustose coralline algae (CCA), and sand potentially associated with microphytobenthos. These categories represented 97 ± 1% of the total seafloor coverage at SHE and 91 ± 2% at EXP and were therefore selected as representative primary producers for the subsequent incubation experiments. During benthic community assessment, rugosity was determined at both sites using the chain method described by Risk (1972). Rugosity was quantified along three 10 m sub-transects within each of the 50 m transects and were used to calculate the rugosity factor for each study site as described by McCormick (1994) (SHE: 1.53 ± 0.12, EXP: 1.32 ± 0.13).

### Sampling of organisms

Specimens of scleractinian corals, macroalgae, algal turfs, and CCA as well as sand samples, from 10 ± 1 m water depth were used for quantification of O_2_ fluxes (see Table S1 for number of replicates). All samples were brought to the water surface in Ziploc^®^ bags and transported directly to the field laboratory. Scleractinian corals of the genera *Montastraea* (including the species *M. faveolata, M. franksi* and *M. annularis*, currently belonging to the genus *Orbicella*; Budd et al., 2012) and *Diploria* (including *D. strigosa*, currently belonging to the genus *Pseudodiploria* Budd et al., 2012) accounted for more than 80% of the total coral cover at the study sites and were therefore used as representative corals in our study. Coral specimens were obtained from the reef using hammer and chisel, fragmented with a multifunction rotary tool (8,200–2/45; mean fragment surface area: 13.16 ± 7.96 cm^2^, Dremel Corp.), and fixed on ceramic tiles using epoxy glue (Giesemann GmbH, Aquascape). After fragmentation, specimens were returned to their natural habitat and left to heal for one week prior to the incubation experiments. Algae of the genus *Dictyota* (mainly *D. bartayresiana*) amounted to nearly 100% of macroalgal cover. Therefore small bushes of *Dictyota* spp. (surface area 1.86 ± 0.88 cm^2^) were used as representatives for macroalgae. Macroalgae were transferred to a storage tank (volume: 500 L in which water was exchanged manually 3–5 times per day and water temperature was within the ranges of incubation experiments; Table 2) one day before incubation experiments and left to heal. All other functional groups were incubated immediately after sampling. Rubble overgrown by algal turfs and CCA served as samples for the respective functional group (surface area covered by the organisms: 15.63 ± 10.80 cm^2^ and 7.48 ± 3.60 cm^2^, respectively). For sand samples, custom-made mini corers with defined surface area (1.20 cm^2^) and sediment core depth (1.0 cm) were used. All necessary permits (DGI-SCI-BEM-00488) were obtained by Instituto de Investigaciones Marinas y Costeras (INVEMAR) in Santa Marta, Colombia which complied with all relevant regulations.

**Table 2:** Water temperature and light intensity during incubation experiments at sampling sites and in incubation containers. All values are in mean ± SD. Data in parentheses represent water temperature and light intensity and at the end of the upwelling event in 2010/2011.

	**Non-upwelling**		**Upwelling**	
	*In situ*	Incubations	*In situ*	Incubations
Temperature (°C)	29.1 ± 0.2	28.6 ± 0.5	25.3 ± 0.3 (26.1 ± 0.2)	25.4 ± 0.6 (26.5 ± 0.4)
Light intensity (PAR µmol photons m^−2^ s^−1^)	146 ± 47	154 ± 40	230 ± 58 (234 ± 78)	257 ± 69 (248 ± 71)

### Surface area quantification

Digital photographs of coral specimens were used to quantify planar projected surface areas of samples by image-processing software (ImageJ, V. 1.46r, National Institute of Health). The 3D surface area of the samples was estimated via multiplication of the planar projected surface areas by the genera-specific 2D to 3D surface area conversion factors derived from computer tomography measurements of *Diploria* and *Montastraea* skeletons (2.28 ± 0.16 and 1.34 ± 0.56, respectively), as described by Naumann et al. (2009). Planar leaf area of spread out macroalgal specimens was likewise quantified by digital image analysis and multiplied by the factor 2 to obtain 3D surface area of the samples. Image analysis of *in situ* photographs and whole spread out macroalgal thalli were used to obtain covered substrate areas (2D surface) as well as 3D surface areas in order to calculate the 2D to 3D conversion factor for macroalgae (4.29 ± 0.82). This conversion factor was used to correct for the overlap of macroalgal tissue. The 2D surface area of algal turf samples was determined by image analysis of digital photographs. For CCA, the simple geometry method described by Naumann et al. (2009) was used to estimate the surface area of overgrown pieces of rubble. The obtained surface areas were related to the planar projected surface area of the samples to generate 2D to 3D conversion factors for CCA (2.10 ± 0.89). Specimen surface area for sand samples was defined by the size of the utilized mini corer (1.20 cm^2^).

## Incubation Experiments

Prior to incubation experiments, water temperature (°C) and light intensity (lx) were monitored at the sampling sites with intervals of 2 min using light and temperature loggers (Onset HOBO Pendant UA-002-64) in order to adjust light and temperature during incubations to *in situ* conditions. The availability of light during light incubations was adjusted to the *in situ* light regimes using net cloth (Table 2). Temperature and light intensity was continuously monitored during incubations as described above. Light intensities were converted to photosynthetically active radiation (PAR, µmol photons m^−2^ s^−1^, 400–700 nm) using the approximation of Valiela (1995). Light availability was generally higher during the upwelling event (*t*-test, *p* < 0.001; Table 2), whereas water temperatures were higher during non-upwelling (*t*-test, *p* < 0.001; Table 2). Quantification of photosynthetic activity for macroalgae, CCA, and microphytobenthos were performed in air-tight glass containers with volumes of 60 mL, whereas for corals and algal turfs, containers with volumes of 600 mL were utilized. For all incubations, we used freshly collected seawater from Gayraca Bay. To ensure independence between the samples, each specimen was incubated in a distinct container. The containers were placed in cooling boxes filled with seawater to maintain constant *in situ* water temperature (Table 2). For dark incubations during daytime, the above mentioned methodology was used, but cooling boxes were closed with opaque lids to prevent light penetration. Comparability among measurements was assured by carrying out all light incubations on cloudless days between 10 am and 2 pm. For each group of primary producers, one light and one dark incubation were performed within each study period. Incubation containers containing only seawater served as blank controls to quantify photosynthetic activity and respiration of microbes in the water column. Physiological damage of the investigated specimens by hypoxic or hyperoxic conditions were prevented by keeping the incubation times as short as possible (light incubations: 30–60 min and dark incubations: 120 min as suggested by Jantzen et al., 2008; Mass et al., 2010b; Jantzen et al., 2013). Dissolved O_2_ concentrations in the incubation water within the glass containers were quantified before incubations and after removing the specimens at the end of each incubation using an optode (Hach Lange, HQ 40). Before O_2_ measurements, the incubation medium was gently stirred with the optode sensor allowing a homogenization of the water column. Experiments were conducted in closed, non-mixed incubation chambers in order to avoid additional contamination sources and to provide the most conservative estimates of O_2_ production rates of benthic primary producers as suggested by Haas et al. (2011) and Naumann et al. (2013). This also ensured higher measurement accuracy, as water movement during incubations may affect gas transfer velocities across the surface boundary of the incubation chambers (Wu, Barazanji & Johnson, 1997) and allowed us to compare our results with previous incubation studies (e.g., Haas et al., 2011; Jantzen et al., 2013; Naumann et al., 2013). Nevertheless, since it is well known that water flow enhances O_2_ fluxes and thereby photosynthesis (Mass et al., 2010a), the results of the field incubations should be regarded as conservative estimates of *in situ* O_2_ fluxes and interpreted accordingly.

### Data analyses and statistics

To quantify net O_2_ production (*P_n_*) and respiration of functional groups, O_2_ concentration before incubations was subtracted from concentration after incubations and blank control values were subtracted from the measured O_2_ fluxes. Individual gross O_2_ production (*P_g_*) of investigated functional groups was calculated by adding values of *P_n_* and respiration; individual O_2_ fluxes were expressed as mmol O_2_ m^−2^ specimen surface area h^−1^.

The contribution of each functional group to total reef production (given as: mmol O_2_ m^−2^ seafloor area h^−1^) was estimated as follows:

\begin{eqnarray*} {c}_{i}={p}_{i}\hspace{0.167em} {s}_{i}\hspace{0.167em} {b}_{i}\hspace{0.167em} r \end{eqnarray*}

taking into account the individual production rates (*p_i_*), the respective mean 2D to 3D surface conversion factor (*s_i_*), group-specific benthic coverage (*b_i_*) as well as the rugosity factor (*r*). Estimation of total daily benthic productivity was furthermore calculated by summing up the contribution of the investigated groups and extrapolating the incubation periods to a 12 h light and 12 h dark cycle.

After testing for normal distribution (Kolmogorov-Smirnoff test) and homogeneity of variances (Levene test), benthic coverage of functional groups were analyzed using two-way ANOVA and Bonferroni’s *post hoc* tests to detect possible effects of season (upwelling vs. non-upwelling) and site (EXP vs. SHE) and their interaction on benthic cover.

We tested the influence of benthic groups, season, wave exposure, and their interactions on O_2_ productivity by generalized linear models (GLMs) for individual *P_n_* and *P_g_* of the investigated groups, their contribution to reef metabolism as well as total benthic productivity. We used Markov-chain Monte Carlo (MCMC) estimations of GLM regression coefficients. In traditional Frequentist statistics, the parameters of interest (i.e., the O_2_ productivity describing regression coefficients) are estimated just once (e.g., using Maximum-Likelihood) and their significance is inferred indirectly based on a test-statistic. In contrast, Bayesian methods reallocate the coefficients across a set of possible candidates during each MCMC generation (Kruschke, 2011). If the bulk of these values, that is the 95% highest posterior density (HPD), does not include zero, one can directly conclude that the regression coefficient is credible different than zero and an effect on O_2_ productivity exists. Moreover, we here performed pair-wise comparisons between benthic groups at different sites and seasons, traditionally being performed by *post-hoc* testing with *P*-value correction for preventing false positive results. A Bayesian GLM does not suffer this drawback because difference of groups can be directly estimated by the posterior (Kruschke, 2011). Again, there is credible evidence in non-equal group-means, if the posterior-based 95% HPD interval of the group differences does not include zero. Model performance for all 19 possible combinations of the three independent variables and their interactions was assessed by the deviance information criterion (DIC), a Bayesian measure of model fit that penalizes complexity (Spiegelhalter et al., 2002). In this information theory based model selection, often there is not a single best model describing the data. Therefore, averaging of regression coefficients for all models within ΔDIC < 2 of the best one (Johnson & Omland, 2004) was performed according to DIC weights (i.e., support for the respective regression model).

Here, Bayesian GLMs using the MCMCglmm package (Hadfield, 2010) for the R 3.0.3 environment for statistical computing (R Core Team, 2014) with a Gaussian error distribution were applied. Prior to the analyses, the mean–variance relationship of measured O_2_ flux was stabilized by power transformation (Yeo & Johnson, 2000). Visual inspection of preliminary GLMs with default weakly informative priors showed high autocorrelation in their posterior distribution. Thus to infer the posterior distribution of the final analyses, we ignored the first 50,000 estimates as burn-in and sampled every 5th out of 650,000 MCMC generations.

All values are represented as mean ± standard deviation (SD) if not noted otherwise.

## Results

### Benthic community composition

**Figure 2:**
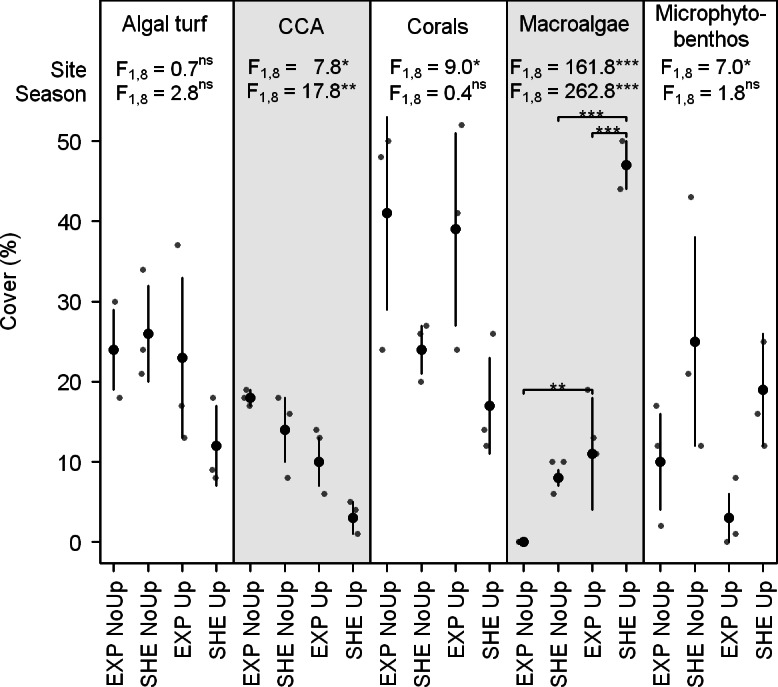
Benthic cover of dominant benthic groups. Jitter plot of grey dots indicates benthic coverage of functional groups quantified by line transects. Superimposed black points and error bars represent the mean ± standard deviation. *F*-values refer to the results of two-way analyses of variance with Site and Season as main effects. If interactions between main effects were significant, pair-wise *post hoc* tests were applied. Significance levels are ^∗^*p* < 0.05, ^∗∗^*p* < 0.01, ^∗∗∗^*p* < 0.001. Abbreviations: EXP, exposed; SHE, sheltered; NoUp, non-upwelling; Up, upwelling.

At EXP, scleractinian corals dominated the benthic community during non-upwelling and upwelling (41 ± 12 and 39 ± 12%, respectively; Fig. 2). At SHE, corals, algal turf, and sand cover was similar during non-upwelling (24 ± 3%, 26 ± 6%, and 25 ± 13%, respectively), while during upwelling, macroalgae exhibited highest benthic cover (47 ± 3%, Fig. 2). During the entire study period, coral and CCA cover was significantly higher at EXP than at SHE, whereas sand showed a contrary pattern with significantly more coverage at SHE (Fig. 2). Macroalgae was the only group where interaction between sites and seasons occurred with significantly higher cover at SHE and higher abundances during upwelling at both sites (Fig. 2). CCA cover also differed between the seasons, showing a significant decrease during the upwelling event (Fig. 2).

### O_2_ fluxes of organisms

More complex Bayesian GLMs, including interactions among the three independent variables season, benthic group, and site, described individual O_2_ fluxes better than simple models (For details see Table S2).

**Figure 3:**
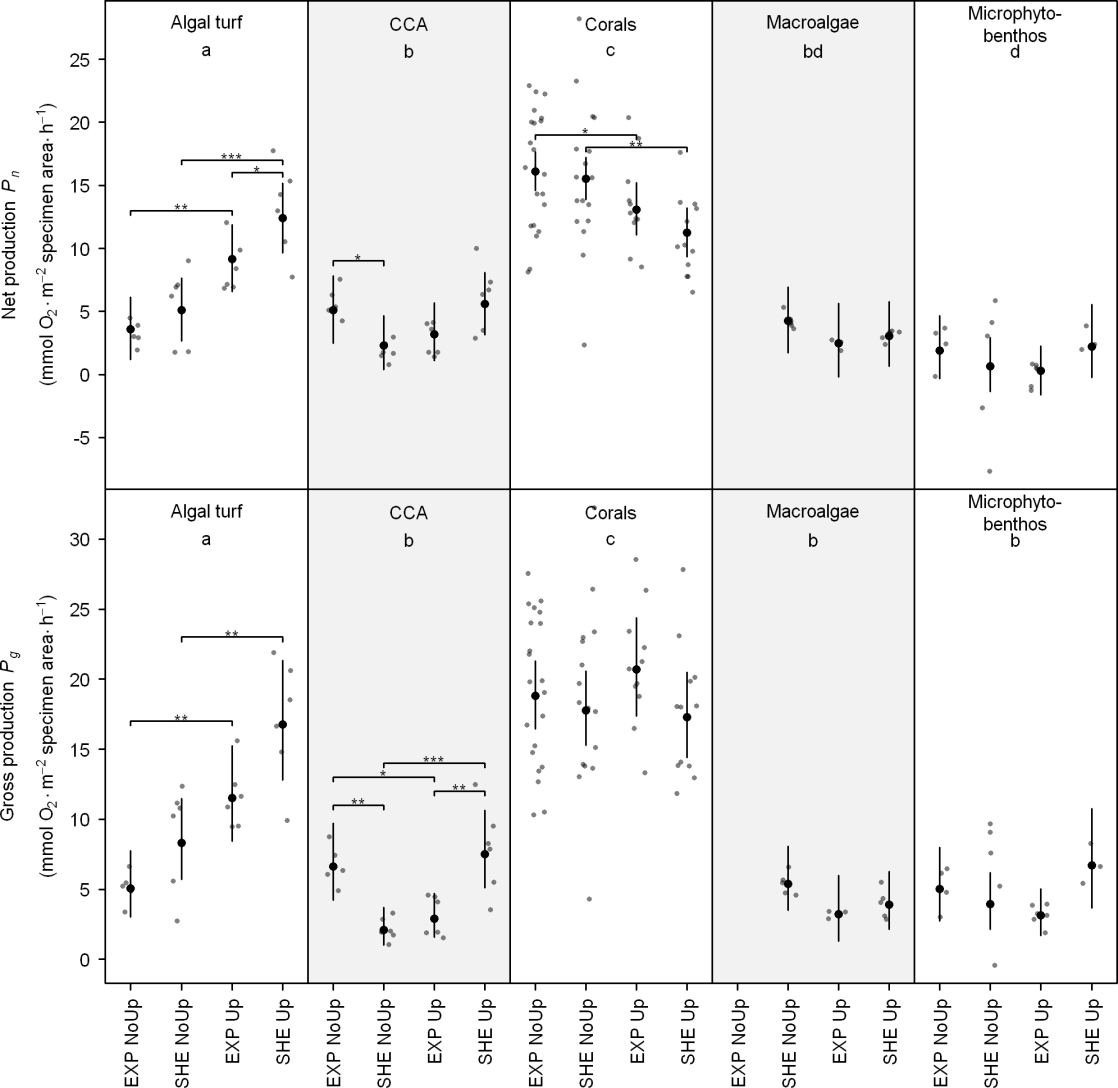
Individual net and gross production of benthic functional groups. Jitter plot of grey dots indicates measured O_2_ fluxes. Superimposed black points and error bars represent the mean and 95% confidence interval according to the Bayesian generalized linear model. Equal lowercase letters indicate no differences in mean productivity among benthic groups and brackets display differences within groups. Significance levels are ^∗^*p*MCMC < 0.05, ^∗∗^*p*MCMC < 0.01, ^∗∗∗^*p*MCMC < 0.001. Abbreviations: EXP, exposed; SHE, sheltered; NoUp, non-upwelling; Up, upwelling.

Of all investigated functional groups, scleractinian corals had highest individual net (*P_n_*) and gross production (*P_g_*), followed by algal turfs, macroalgae, CCA, and microphytobenthos (Fig. 3; see also Table S3 for detailed results of all pair-wise comparisons). Regarding spatial differences in individual productivity, significant differences were detected for algal turfs and CCA. During upwelling, *P_n_* of algal turfs and *P_g_* of CCA was higher at SHE than EXP. On the contrary, during non-upwelling, *P_n_* and *P_g_* of CCA was higher at EXP (Fig. 3).

Temporal differences in O_2_ production were detected for corals, algal turfs, and CCA (Fig. 3). Whereas *P_n_* of scleractinian corals on both sites and *P_g_* of CCA at EXP were higher during non-upwelling, *P_g_* of CCA at SHE as well as *P_n_* and *P_g_* of algal turfs at both sites showed an opposite pattern with higher productivity rates during upwelling (Fig. 3).

### Contribution of organism-induced O_2_ fluxes to total reef O_2_production

As in the case of individual O_2_ fluxes, contribution and total reef production were better explained by GLMs of higher complexity (Table S2).

**Figure 4:**
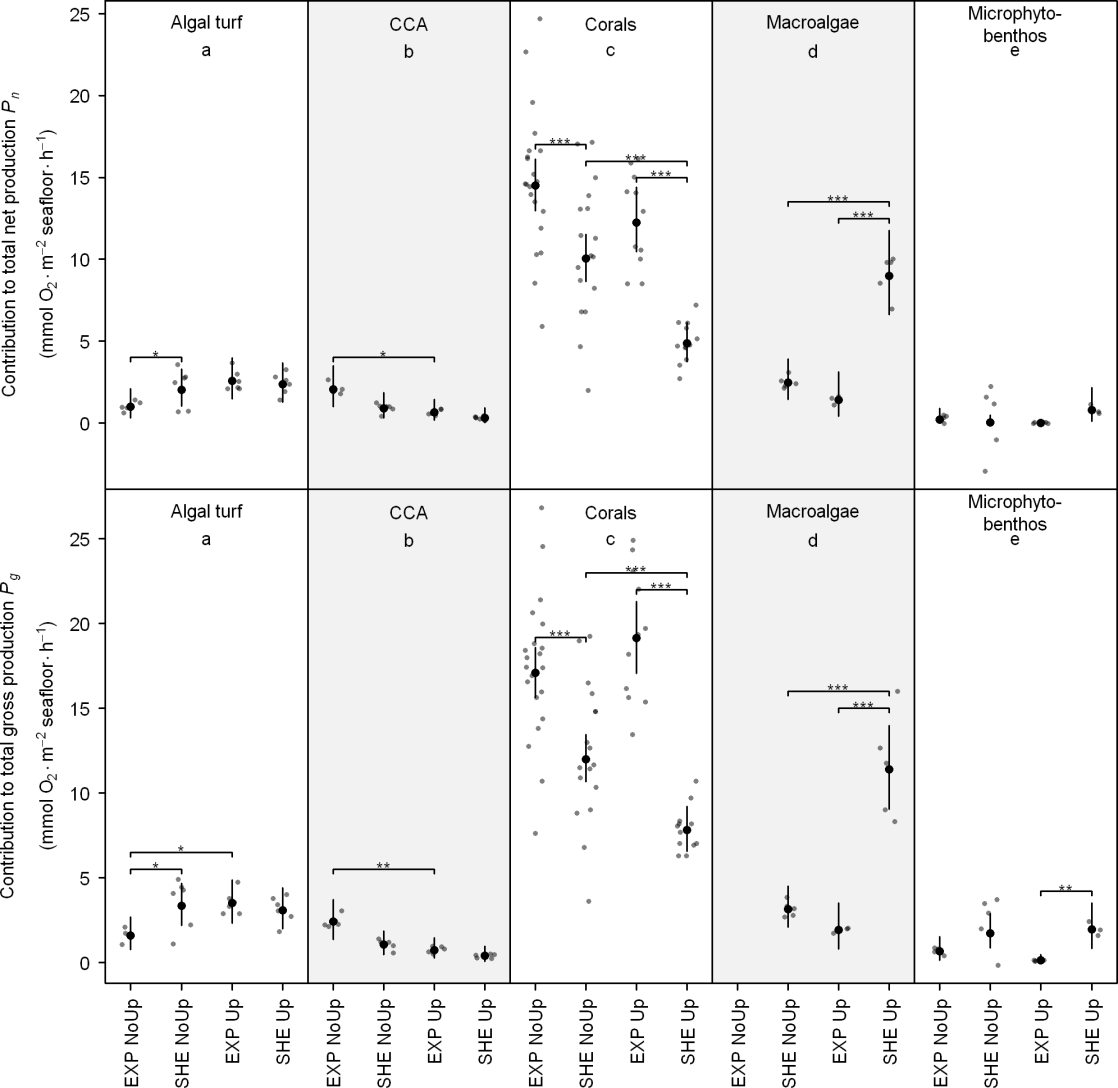
Contribution of functional groups to benthic net and gross production. Jitter plot of grey dots indicates measured O_2_ fluxes. Superimposed black points and error bars represent the mean and 95% confidence interval according to the Bayesian generalized linear model. Equal lowercase letters indicate no differences in mean productivity among benthic groups and brackets display differences within groups. Significance levels are ^∗^*p*MCMC < 0.05, ^∗∗^*p*MCMC < 0.01, ^∗∗∗^*p*MCMC < 0.001. Abbreviations: EXP, exposed; SHE, sheltered; NoUp, non-upwelling; Up, upwelling.

Contribution of functional groups to benthic productivity exhibited similar pattern than individual productivity with corals contributing generally most to total reef *P_n_* and *P_g_*, but macroalgae contributed most to benthic *P_n_* and *P_g_* at SHE at the end of upwelling (Fig. 4; see also Table S3 for detailed results of all pair-wise comparisons).

Significant spatial differences in contribution to total benthic *P_n_* within functional groups were detected for corals, algal turf, and macroalgae, and spatial differences for *P_g_* were present in all investigated groups except CCA (Fig. 4). At EXP, Corals contributed more to total *P_n_* and *P_g_* during non-upwelling and upwelling (Fig. 4). At SHE, contributions of macroalgae (*P_n_* and *P_g_*) and microphytobenthos (*P_g_*) were higher only during upwelling, and algal turfs contributed more to *P_g_* at SHE during non-upwelling (Fig. 4).

**Figure 5:**
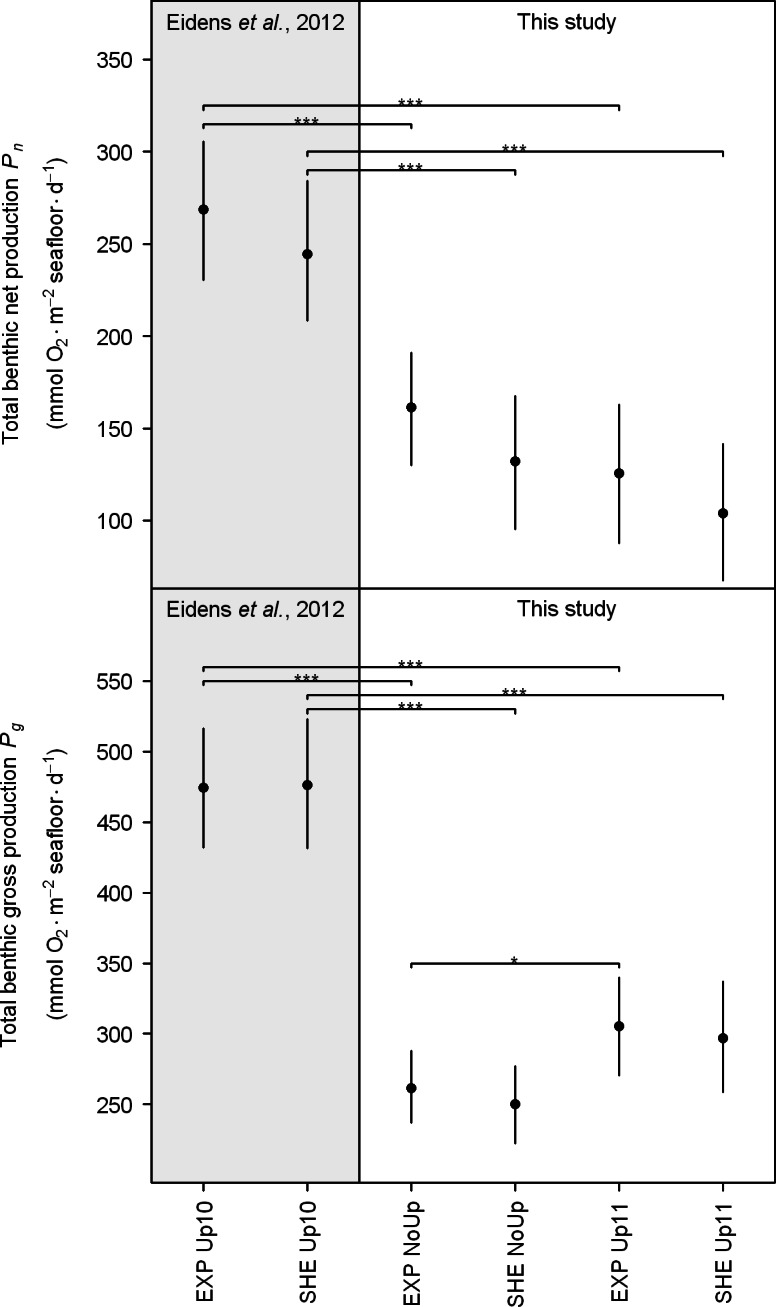
Total benthic net and gross production. Black points and error bars represent the mean and 95% confidence interval according to the Bayesian generalized linear model. Brackets display differences between seasons. Significance levels are ^∗^*p*MCMC < 0.05, ^∗∗^*p*MCMC < 0.01, ^∗∗∗^*p*MCMC < 0.001. Abbreviations: EXP, exposed; SHE, sheltered; NoUp, non-upwelling; Up10, upwelling 2010/2011; Up11, upwelling 2011/2012.

Temporal differences in contribution to total benthic productivity within the investigated groups were present for corals, macroalgae, CCA (for *P_n_* and *P_g_*), and for algal turfs (only *P_g_*) (Fig. 5). During non-upwelling, corals contributed more to the total productivity at SHE and CCA at EXP, whereas during upwelling, macroalgae contributed more to the total productivity at SHE and algal turf at EXP (Fig. 4).

Regarding the total daily benthic O_2_ fluxes (Fig. 4), no spatial differences between EXP and SHE were detected, neither during non-upwelling nor during upwelling (see also Table S3 for detailed results of all pair-wise comparisons). During the study, significant temporal differences were only present for *P_g_* at the exposed site with higher O_2_ fluxes during the upwelling in 2011/2012 compared to non-upwelling (Fig. 5). Comparing total benthic productivity during the upwelling event in 2010/2011 with the subsequent non-upwelling and upwelling, *P_n_* and *P_g_* were significantly higher during the upwelling 2010/2011 for all comparisons (Fig. 5).

## Discussion

### O_2_ fluxes of organisms

Individual mean *P_n_* and *P_g_* were generally highest for corals at both sites during the study periods (*P_n_*: 11.2–16.1 and *P_g_*: 17.4–20.8 mmol O_2_ m^−2^ specimen area h^−1^). These high productivity rates of corals compared to other investigated primary producers (see Fig. 3) may be attributed to the mutualistic relationship between zooxanthellae and coral host leading to enhanced photosynthetic efficiency under high CO_2_ and nutrient availability (D’Elia & Wiebe, 1990; Muscatine, 1990). Estimated daily *P_g_* per m^2^seafloor for the investigated coral genera, (441–610 mmol O_2_ m^−2^ seafloor d^−1^), is within the range of other Caribbean corals (67–850 mmol O_2_ m^−2^ seafloor d^−1^, Fig. 2, Kanwisher & Wainwright, 1967), and O_2_ fluxes of all investigated organism groups are comparable to values reported in the literature (Fig. 2).

Significant spatial differences during non-upwelling were found for CCA with higher productivity at EXP compared to SHE (Fig. 3). These differences may be attributed to the prevailing water current regime in the bay together with high water temperatures during non-upwelling (Tables 1 and 2). An increase in water temperature typically intensifies metabolic activity in CCA (Hatcher, 1990; Littler & Doty, 1975). However, the lower water flow at SHE (Bayraktarov, Bastidas-Salamanca & Wild, 2014) may have prevented the required gas exchange and nutrient uptake, resulting in lower individual CCA productivity at this site. In contrast, the higher rates in individual productivity of algal turfs and CCA at SHE during upwelling (Fig. 3) are potentially a result of the differences in species composition (sensu Littler, 1973; Chisholm, 2003; Copertino, Cheshire & Kildea, 2009; Ferrari et al., 2012).

Temporal differences in individual O_2_ production within the investigated organism groups generally showed two contrary patterns: whereas scleractinian corals on both sites and CCA at EXP produced less O_2_ during upwelling, algal turfs and CCA at SHE produced more O_2_. The decreased productivity rates of corals and CCA at EXP during upwelling indicate that low water temperature has an adverse effect on the productivity of these groups. This argument is supported by studies showing that low water temperatures lead to a decrease in photosynthetic performance of primary producers in coral reefs (Hatcher, 1990; Kinsey, 1985). In contrast, the two-fold higher photosynthetic performance of algal turfs during upwelling may be due to higher nutrient concentrations together with higher water currents during this season (Bayraktarov, Bastidas-Salamanca & Wild, 2014; Bayraktarov, Pizarro & Wild, 2014), facilitating gas exchange and nutrient uptake. Our findings are supported by Carpenter & Williams (2007), showing that photosynthesis of algal turfs in coral reefs is mainly limited by nutrient uptake, which in turn depends on nutrient availability and water current speed. Whereas productivity of CCA at EXP seems to be temperature-limited, our findings indicate that their productivity at SHE is limited by nutrient availability as previously suggested for benthic algal communities in water current-sheltered coral reef locations (Carpenter & Williams, 2007; Hatcher, 1990).

### Contribution of organism-induced O_2_ fluxes to total benthic O_2_ production

Our results indicate that the spatial differences in contribution to total benthic O_2_ production for scleractinian corals, macroalgae, CCA, and microphytobenthos are directly linked to spatial differences in their benthic coverage. For instance, the major contribution of corals (Fig. 4) can be explained by their comparably high benthic coverage (ranging from 24 to 39%; Fig. 2) and highest quantified individual O_2_ production rates among all investigated groups (Fig. 3). This finding is supported by the estimates of Wanders (1976a), showing that corals accounted for about two-thirds of the total benthic primary production in a Southern Caribbean fringing reef.

Although individual macroalgal production rates were rather low as compared to coral productivity (Fig. 3), the extremely high cover of macroalgae at SHE during upwelling (47 ± 3%, Fig. 2) resulted in macroalgae being the main contributors to total benthic production. Macroalgal cover (incl. the dominant genus *Dictyota*) has previously been found to be particularly high during upwelling (Bula-Meyer, 1990; Cronin & Hay, 1996; Diaz-Pulido & Garzón-Ferreira, 2002) probably due to elevated nutrient concentrations and low water temperatures (Bayraktarov, Pizarro & Wild, 2014).

The elevated contributions of corals and CCA at EXP as well as macroalgae and microphytobenthos at SHE during upwelling (Fig. 4) might be due to site-specific differences in abundances (Fig. 2), which in turn are likely caused by site-specific differences in water current regimes (Bayraktarov, Bastidas-Salamanca & Wild, 2014; Werding & Sánchez, 1989).

Corals, macroalgae, algal turfs, and CCA also exhibited distinct temporal differences in contribution to total benthic productivity. At SHE, corals contributed more to the benthic O_2_ production during non-upwelling and macroalgae and algal turfs during upwelling, whereas contribution of CCA at EXP was higher during non-upwelling. These differences can be explained with seasonal growth patterns, temperature-dependent changes in individual O_2_ productivity and temporal shifts in abundances (Figs. 2 and 3). Opposite abundance patterns of CCA and macroalgae are, for example, in agreement with previous studies showing that macroalgae can shade CCA, usually leading to negative correlated abundances of these groups (Belliveau & Paul, 2002; Lirman & Biber, 2000).

### Total benthic O_2_ fluxes and ecological perspective

The estimated total daily benthic O_2_ production at both sites during non-upwelling and upwelling (Fig. 5) are, although comparable, on average slightly lower than the values previously reported for other fore reefs communities (Table 3). These differences might be due to a methodological bias. Whereas previous studies utilized flow respirometry techniques, the current study used incubation methodology, which accounts for production values in the target groups only.

**Table 3:** Mean benthic oxygen production of reef communities and their dominant functional groups of primary producers. If necessary, original units were converted to O_2_ estimates assuming a C:O_2_ metabolic quotient equal to one according to Gattuso et al. (1996) and Carpenter & Williams (2007).

	Location	*P_n_*	*P_g_*	Reference
		(mmol O_2_ m^−2^ seafloor d^−1^)		
**Reef slope/fore reef communities**	Caribbean	103–169	250–305	This study
	Caribbean	125–272	250–483	Eidens et al. (2012)
	Various Atlantic/Pacific	−83–425	167–583	Hatcher (1988)
	Caribbean	113–469	313–638	Adey & Steneck (1985)
**Functional group**				
Corals	Caribbean	227–344	441–610	This study
	Caribbean	328–369	441–598	Eidens et al. (2012)
	Caribbean	166	447	Wanders (1976a)
	Caribbean		225–850	Kanwisher & Wainwright (1967)
Macroalgae	Caribbean	117–244	198–375	This study
	Caribbean	244–444	375–624	Eidens et al. (2012)
	Caribbean	142–433	250–633	Wanders (1976b)
	Various Atlantic/Pacific		192–3283	Hatcher (1988)
Algal turfs	Caribbean	39–157	84–253	This study
	Caribbean	39–339	84–554	Eidens et al. (2012)
	Various Atlantic/Pacific		75–1008	Hatcher (1988)
	Various Atlantic/Pacific		83–967	Kinsey (1985)
	Caribbean	175–433	308–617	Wanders (1976a)
Crustose coralline algae	Caribbean	44–104	58–140	This study
	Caribbean	44–104	58–140	Eidens et al. (2012)
	Various Atlantic/Pacific		67–83	Kinsey (1985)
	Caribbean	58–117	192–258	Wanders (1976a)
	Great Barrier Reef	50–333	75–416	Chisholm (2003)
Microphytobenthos	Caribbean	1–67	75–143	This study
	Caribbean	6–87	78–191	Eidens et al. (2012)
	SW Pacific	0–8	92–150	Boucher et al. (1998)
	Various Atlantic/Pacific		50–225	Kinsey (1985)

**Notes.**

Pn net O_2_ production Pg gross O_2_ production

Despite the high spatial and temporal differences in benthic coverage and group-specific O_2_ fluxes of investigated benthic primary producers as well as their contribution to total benthic productivity, no spatial differences in total benthic O_2_ fluxes were detected between EXP and SHE. These results were consistent during both non-upwelling and upwelling (Fig. 5). Our findings are supported by Hatcher (1990), showing that the relative coverage of benthic photoautotrophs in a reef community may have little effect on its areal production rate. In TNNP, seasonal differences were only present for *P_g_* at EXP with higher rates during upwelling compared to non-upwelling. These differences are mainly related to individual productivity of algal turfs, being generally two-fold higher during upwelling compared to non-upwelling (Fig. 3), and to the absence of macroalgae at EXP during non-upwelling (Fig. 2). This is in agreement with studies by Kinsey (1985) and Hatcher (1990), reporting that algae, as one of the most seasonal component in coral reefs, account for seasonal shifts in benthic reef productivity.

The lack of seasonality of *P_n_* and *P_g_* regarding communities at SHE as well as *P_g_* at EXP stands in contrast to earlier studies (Eidens et al., 2012; Kinsey, 1977; Kinsey, 1985; Smith, 1981), which found an approximately two-fold difference in benthic primary production between seasons. This lack of seasonality in *P_n_* and partly in *P_g_* might be related to seasonal changes of abiotic factors in TNNP that compensate for each other (Table 1). The observed similarity in productivity rates during different seasons suggest that coral reefs in TNNP can cope with pronounced seasonal variations in light availability, water temperature, and nutrient availability. Nevertheless, total *P_n_* and *P_g_* during the upwelling in 2010/2011 (*P_n_*: 244–272 and *P_g_*: 476–483 mmol O_2_ m^−2^ seafloor d^−1^) were not only higher compared to non-upwelling (Eidens et al., 2012) but also higher than during the subsequent upwelling in 2011/2012 (Fig. 5). These findings suggest that interannual variations affect the productivity of TNNP coral reefs. Dramatic ENSO-related water temperature increases and high precipitation in the study area (Bayraktarov et al., 2013; Hoyos et al., 2013) led to coral bleaching at the end of 2010 (Bayraktarov et al., 2013). Surprisingly, bleached corals in the bay recovered quickly in the course of the following upwelling event (Bayraktarov et al., 2013) and exhibited similar O_2_ production rates during all study periods (Eidens et al., 2012), indicating a high resilience of TNNP corals. Moreover, macroalgae and algal turf seemed to benefit from the environmental conditions during the upwelling following the ENSO-related disturbance events, resulting in higher group-specific productivity during the upwelling in 2010/2011 compared to subsequent study periods (Eidens et al., 2012). The elevated production rates of macroalgae and algal turfs together with the quick recovery of corals from bleaching likely accounted for a higher benthic productivity during the upwelling in 2011/2011 compared to non-upwelling (Eidens et al., 2012) and the upwelling in 2011/2012 (Fig. 5). These findings indicate that extreme ENSO-related disturbances do not have long-lasting effects on the functioning of local benthic communities in TNNP.

In conclusion, the present study showed that total benthic productivity in TNNP is relatively constant despite high variations in key environmental parameters. This stable benthic productivity suggests a relatively high resilience of local benthic communities against natural environmental fluctuations and anthropogenic disturbances. We therefore recommend that TNNP should be considered as a conservation priority area.

## Supplemental Information

10.7717/peerj.554/supp-1 Table S1 Raw values of oxygen fluxes in Gayraca Bay

10.7717/peerj.554/supp-2 Table S2 Goodness of fit for generalized linear models Goodness of fit for all 19 general linear models including the three independent variables benthic group, season, and site and their interactions. Dependent variables have been individual net and gross production, contribution of functional groups to benthic net and gross production, and total daily benthic net and gross production, respectively. Grey box indicates inclusion of the independent variable in the respective model. Abbreviations: DIC, deviance information criterion delta; DIC, difference in DIC compared to the model with highest fit; DICwt, DIC weights, i.e., the support for respective the mode.

10.7717/peerj.554/supp-3 Table S3 Pair-wise comparisons of productivity Pair-wise comparisons between means and sums of functional groups as estimated by the posterior distribution of Bayesian generalized linear models. Abbreviations: EXP, exposed; SHE, sheltered; NoUp, non-upwelling; Up, upwelling; Lower HPD, lower 95% confidence interval of highest posterior density; Upper HPD, upper 95% confidence interval of highest posterior density.

## Additional Information and Declarations

### Field Study Permissions

The following information was supplied relating to field study approvals (i.e., approving body and any reference numbers): All necessary permits (DGI-SCI-BEM-00488) were obtained by Instituto de Investigaciones Marinas y Costeras (INVEMAR) in Santa Marta, Colombia which complied with all relevant regulations.

## References

[ref-1] Achituv Y, Dubinsky Z, Dubinsky Z (1990). Evolution and zoogeography of coral reefs. Coral reefs ecosystems of the world.

[ref-2] Adey W, Steneck R, Reaka M (1985). Highly productive eastern Caribbean reefs: synergistic effects of biological, chemical, physical, and geological factors. The ecology of deep and shallow coral reefs.

[ref-3] Andrade CA, Barton ED (2005). The Guajira upwelling system. Continental Shelf Research.

[ref-4] Andrews JC, Pickard GL, Dubinsky Z (1990). The physical oceanography of coral-reef systems. Coral reefs.

[ref-5] Bayraktarov E, Bastidas-Salamanca M, Wild C (2014). The physical environment in coral reefs of the Tayrona National Natural Park (Colombian Caribbean) in response to seasonal upwelling. Boletín de Investigaciones Marinas y Costeras - Invemar.

[ref-6] Bayraktarov E, Pizarro V, Eidens C, Wilke T, Wild C (2013). Bleaching susceptibility and recovery of Colombian Caribbean corals in response to water current exposure and seasonal upwelling. PLoS ONE.

[ref-7] Bayraktarov E, Pizarro V, Wild C (2014). Spatial and temporal variability of water quality in the coral reefs of Tayrona National Natural Park, Colombian Caribbean. Environmental Monitoring and Assessment.

[ref-8] Belliveau SA, Paul VJ (2002). Effects of herbivory and nutrients on the early colonization of crustose coralline and fleshy algae. Marine Ecology-Progress Series.

[ref-9] Boucher G, Clavier J, Hily C, Gattuso J-P (1998). Contribution of soft-bottoms to the community metabolism (primary production and calcification) of a barrier reef flat (Moorea, French Polynesia). Journal of Experimental Marine Biology and Ecology.

[ref-10] Budd AF, Fukami H, Smith ND, Knowlton N (2012). Taxonomic classification of the reef coral family Mussidae (Cnidaria: Anthozoa: Scleractinia). Zoological Journal of the Linnean Society.

[ref-11] Bula-Meyer G (1990). Altas temperaturas estacionales del agua como condición disturbadora de las macroalgas del Parque Nacional Natural Tairona, Caribe colombiano: una hipótesis. Anales del Instituto de Investigaciones Marinas y Costera.

[ref-12] Carpenter RC, Williams SL (2007). Mass transfer limitation of photosynthesis of coral reef algal turfs. Marine Biology.

[ref-13] Chapin FS, Matson PA, Vitousek PM, Chapin MC (2011). Principles of terrestrial ecosystem ecology.

[ref-14] Chisholm JRM (2003). Primary productivity of reef-building crustose coralline algae. Limnology and Oceanography.

[ref-15] Coles S, Fadlallah Y (1991). Reef coral survival and mortality at low temperatures in the Arabian Gulf: new species-specific lower temperature limits. Coral Reefs.

[ref-16] Coles DW, Jokiel P, Connell DW, Hawker DW (1992). Effects of salinity on coral reefs. Pollution in tropical aquatic systems.

[ref-17] Copertino MS, Cheshire A, Kildea T (2009). Photophysiology of a turf algal community: integrated responses to ambient light and standing biomass. Journal of Phycology.

[ref-18] Cortés C, Jiménez C, Cortés J (2003). Past, present and future of the coral reefs of the Caribbean coast of Costa Rica. Latin American coral reefs.

[ref-19] Cronin G, Hay ME (1996). Effects of light and nutrient availability on the growth, secondary chemistry, and resistance to herbivory of two brown seaweeds. Oikos.

[ref-20] Dana FD (1843). On the temperature limiting the distribution of corals. American Journal of Science.

[ref-21] Darwin C (1842). The structure and distribution of coral reefs.

[ref-22] D’Elia CF, Wiebe WJ, Dubinsky Z (1990). Biogeochemical nutrient cycles in coral-reef ecosystems. Coral reefs.

[ref-23] Diaz-Pulido G, Garzón-Ferreira J (2002). Seasonality in algal assemblages on upwelling-influenced coral reefs in the Colombian Caribbean. Botanica Marina.

[ref-24] Eidens C, Bayraktarov E, Pizarro V, Wilke T, Wild C, Yellowlees D, Hughes TP (2012). Seasonal upwelling stimulates primary production of Colombian Caribbean coral reefs.

[ref-25] Falkowski PG, Jokiel PL, Kinzie RA, Dubinsky Z (1990). Irradiance and corals. Coral reefs.

[ref-26] Falter JL, Lowe RJ, Atkinson MJ, Cuet P (2012). Seasonal coupling and de-coupling of net calcification rates from coral reef metabolism and carbonate chemistry at Ningaloo Reef, Western Australia. Journal of Geophysical Research-Oceans.

[ref-27] Ferrari R, Gonzalez-Rivero M, Ortiz JC, Mumby PJ (2012). Interaction of herbivory and seasonality on the dynamics of Caribbean macroalgae. Coral Reefs.

[ref-28] Garzón-Ferreira J, Kjerfve B (1998). Bahía Chengue, Parque Natural Tayrona, Colombia. CARICOMP-Caribbean coral reef, seagrass and mangrove sites Coastal Region and Small Islands Papers 3.

[ref-29] Garzón-Ferreira J, Cano M (1991). Tipos, distribución, extensión y estado de conservación de los ecosistemas marinos costeros del Parque Nacional Natural Tayrona.

[ref-30] Gattuso JP, Pichon M, Delesalle B, Canon C, Frankignoulle M (1996). Carbon fluxes in coral reefs. 1. Lagrangian measurement of community metabolism and resulting air-sea CO_2_ disequilibrium. Marine Ecology-Progress Series.

[ref-31] Gattuso J-P, Frankignoulle M, Wollast R (1998). Carbon and carbonate metabolism in coastal aquatic ecosystems. Annual Review of Ecology and Systematics.

[ref-32] Geyer O (1969). Vorläufige Liste der scleractinen Korallen der Bahía de Concha bei Santa Marta, Kolumbien. Mitteilungen aus dem Instituto Colombo-Alemán de Investigaciones Científicas Punta de Betín.

[ref-33] Glynn PW (1993). Monsoonal upwelling and episodic *Acanthaster* predation as possible controls of coral reef distribution and community structure in Oman, Indian Ocean. Atoll Research Bulletin.

[ref-34] Glynn PW, Stewart RH (1973). Distribution of coral reefs in Pearl Islands (Gulf of Panama) in relation to thermal conditions. Limnology and Oceanography.

[ref-35] Haas AF, Nelson CE, Kelly LW, Carlson CA, Rohwer F, Leichter JJ, Wyatt A, Smith JE (2011). Effects of coral reef benthic primary producers on dissolved organic carbon and microbial activity. PLoS ONE.

[ref-36] Hadfield JD (2010). MCMC methods for multi-response generalized linear mixed models: the MCMCglmm R Package. Journal of Statistical Software.

[ref-37] Hatcher BG (1988). Coral reef primary productivity: a beggar’s banquet. Trends in Ecology & Evolution.

[ref-38] Hatcher BG (1990). Coral reef primary productivity: a hierarchy of pattern and process. Trends in Ecology & Evolution.

[ref-39] Herrmann R (1970). Deutungsversuch der Entstehung der “Brisa”, eines föhnartigen Fallwindes der nordwestIichen Sierra Nevada de Santa Marta, Kolumbien. Mitteilungen des Instituto Colombo-Alemán de Investigaciones Cientificas “Punta de Betin”.

[ref-40] Hodgson G, Kiene W, Mihaly J, Liebeler J, Shuman C, Maun L (2004). Reef check instruction manual: a guide to reef check coral reef monitoring. Reef check.

[ref-41] Hoyos N, Escobar J, Restrepo JC, Arango AM, Ortiz JC (2013). Impact of the 2010–2011 La Niña phenomenon in Colombia, South America: the human toll of an extreme weather event. Applied Geography.

[ref-42] Hubbard DK, Birkeland C (1996). Reefs as dynamic systems. Life and death of coral reefs.

[ref-43] INVEMAR (2012). Sistema de información ambiental marina de Colombia (SIAM).

[ref-44] Jantzen C, Schmidt GM, Wild C, Roder C, Khokiattiwong S, Richter C (2013). Benthic reef primary production in response to large amplitude internal waves at the Similan Islands (Andaman Sea, Thailand). PLoS ONE.

[ref-45] Jantzen C, Wild C, El-Zibdah M, Roa-Quiaoit HA, Haacke C, Richter C (2008). Photosynthetic performance of giant clams, *Tridacna maxima and T. squamosa*, Red Sea. Marine Biology.

[ref-46] Johnson JB, Omland KS (2004). Model selection in ecology and evolution. Trends in Ecology & Evolution.

[ref-47] Kanwisher JW, Wainwright SA (1967). Oxygen balance in some reef corals. Biological Bulletin.

[ref-48] Kinsey DW, Taylor DL (1977). Seasonality and zonation in coral reef productivity and calcification.

[ref-49] Kinsey DW, Gabrie C, Salvat B (1985). Metabolism, calcification and carbon production: 1 systems level studies.

[ref-50] Kleypas JA, McManus JW, Menez LAB (1999). Environmental limits to coral reef development: where do we draw the line?. American Zoologist.

[ref-51] Kruschke JK (2011). Doing Bayesian data analysis: a tutorial with R and BUGS.

[ref-52] Lirman D, Biber P (2000). Seasonal dynamics of macroalgal communities of the northern Florida reef tract. Botanica Marina.

[ref-53] Littler MM (1973). The productivity of Hawaiian fringing-reef crustose Corallinaceae and an experimental evaluation of production methodology. Limnology and Oceanography.

[ref-54] Littler MM, Doty MS (1975). Ecological components structuring seaward edges of tropical pacific reefs: distribution, communities and productivity of *Porolithon*. Journal of Ecology.

[ref-55] Mass T, Genin A, Shavit U, Grinstein M, Tchernov D (2010a). Flow enhances photosynthesis in marine benthic autotrophs by increasing the efflux of oxygen from the organism to the water. Proceedings of the National Academy of Sciences of the United States of America.

[ref-56] Mass T, Kline DI, Roopin M, Veal CJ, Cohen S, Iluz D, Levy O (2010b). The spectral quality of light is a key driver of photosynthesis and photoadaptation in Stylophora pistillata colonies from different depths in the Red Sea. Journal of Experimental Biology.

[ref-57] McCormick MI (1994). Comparison of field methods for measuring surface-topography and their associations with a tropical reef fish assemblage. Marine Ecology-Progress Series.

[ref-58] Muscatine L, Dubinsky Z (1990). The role of symbiotic algae in carbon and energy flux in reef corals. Coral reefs.

[ref-59] Naumann MS, Jantzen C, Haas AF, Iglesias-Prieto R, Wild C (2013). Benthic primary production budget of a caribbean reef lagoon (Puerto Morelos, Mexico). PLoS ONE.

[ref-60] Naumann MS, Niggl W, Laforsch C, Glaser C, Wild C (2009). Coral surface area quantification-evaluation of established techniques by comparison with computer tomography. Coral Reefs.

[ref-61] Odum HT, Odum EP (1955). Trophic structure and productivity of a windward coral reef community on Eniwetok Atoll. Ecological Monographs.

[ref-62] R Core Team (2014). R: a language and environment for statistical computing.

[ref-63] Risk MJ (1972). Fish diversity on a coral reef in the Virgin Islands. Atoll Research Bulletin.

[ref-64] Rueda-Roa DT, Muller-Karger FE (2013). The southern Caribbean upwelling system: sea surface temperature, wind forcing and chlorophyll concentration patterns. Deep-Sea Research Part I-Oceanographic Research Papers.

[ref-65] Salzwedel H, Müller K (1983). A summary of meteorological and hydrological data from the Bay of Santa Marta, Colombian Caribbean. Anales del Instituto de Investigaciones Marinas de Punta de Betín.

[ref-66] Sargent M, Austin T (1949). Organic productivity of an atoll. Transactions of the American Geophysical Union.

[ref-67] Sheppard C, Davy SK, Pilling GM (2009). The biology of coral reefs.

[ref-68] Smith SV (1981). The Houtman Abrolhos Islands: carbon metabolism of coral reefs at high-latitude. Limnology and Oceanography.

[ref-69] Spiegelhalter DJ, Best NG, Carlin BP, Van Der Linde A (2002). Bayesian measures of model complexity and fit. Journal of the Royal Statistical Society: Series B (Statistical Methodology).

[ref-70] Steneck RS, Choat JH, Barnes D, Borowitzka MA, Coll JC, Davies PJ, Flood P, Hatcher BG, Hopley D, Hutchings PA, Kinsey D, Orme GR, Pichon M, Sale PF, Sammarco P, Wallace CC, Wilkinson C, Wolanski E, Bellwood O (1988). Herbivory on coral reefs: a synthesis.

[ref-71] Szmant AM, Lessios HA, Macintyre IG (1997). Nutrient effects on coral reefs: a hypothesis on the importance of topographic and trophic complexity to reef nutrient dynamics.

[ref-72] Valiela I (1995). Marine ecological processes.

[ref-73] Veron JEN (1995). Corals in space and time: biogeography and evolution of the Scleractinia.

[ref-74] Wanders JBW (1976a). The role of benthic algae in the shallow reef of Curaçao (Netherlands Antilles). I: primary productivity in the coral reef. Aquatic Botany.

[ref-75] Wanders JBW (1976b). The role of benthic algae in the shallow reef of Curacao (Netherlands Antilles) II: primary productivity of the Sargassum beds on the north-east coast submarine plateau. Aquatic Botany.

[ref-76] Werding B, Sánchez H (1989). The coral formations and their distributional pattern along a wave exposure gradient in the area of Santa Marta, Colombia. Medio Ambiente.

[ref-77] Wu EY, Barazanji KW, Johnson RLJ (1997). Source of error on A-aDO2 calculated from blood stored in plastic and glass syringes. Journal of Applied Physiology.

[ref-78] Yeo I-K, Johnson RA (2000). A new family of power transformations to improve normality or symmetry. Biometrika.

